# Effects of Fatigue and Grit on Club Sports Coaches

**DOI:** 10.3390/ijerph18147414

**Published:** 2021-07-11

**Authors:** Alfonso Martínez-Moreno, Francisco Cavas-García, José María López-Gullón, Arturo Díaz-Suárez

**Affiliations:** Department of Physical Activity and Sports, CEIR Campus Mare Nostrum (CMN), University of Murcia, Santiago de la Ribera, 30720 San Javier, Spain; francisco.cavas@um.es (F.C.-G.); luchamurcia@um.es (J.M.L.-G.); ardiaz@um.es (A.D.-S.)

**Keywords:** coaches, perseverance, passion, fatigue, motivation, workers

## Abstract

The objective of this research is to identify the level of general fatigue (FG), physical fatigue (FF) and concentration/motivation (C/M) in sports coaches. Two components of grit, consistency of interest (CI) and perseverance in effort (PE), are also assessed. The possible effects of sex, age, marital status, employment contract, work dedication and grit on FG, FF and C/M in sports coaches are examined. This cross-sectional study analyses 335 sports club coaches (21.2% women, 78.8% male) with a mean age of 29.88 (SD = 9.97) years, at a significance level of *p* < 0.05 for all analyses. Different aspects of fatigue were determined using the Spanish translation of the Multidimensional Fatigue Inventory-20 (IMF-20). The Grit-S scale was used to measure the ability to persevere, have passion and commit. The results indicated that men scored higher in FF, C/M and PE, while women obtained higher values in FG and CI. Non-contract coaches had higher FG, CI and PE, while coaches with contracts scored higher on C/M and FF. In conclusion, coaches with higher CI had higher FG, and high levels of PE were associated with low FG levels.

## 1. Introduction

Fatigue is a multifactorial phenomenon that is associated with physical, socioeconomic and environmental risk factors [1], is observed worldwide, and represents a direct and indirect economic/social cost to organisations and countries [2].

The symptom of fatigue is a clinical finding that correlates with various pathologies and locations in the central nervous system [3]. Clinically, fatigue consists of two components. The first is a physical component, indicated by difficulty starting an activity (without objective findings) and a decreased ability to maintain the activity. The second component encompasses difficulties with concentration and memory as well as emotional exhaustion [4]. Additionally, fatigue directly undermines an individual’s quality of life and has been associated with negative health-related outcomes [5].

In this article, we view fatigue as a multidimensional construct that can be classified into neuromuscular (or physical), mental (or cognitive) and visual (or perceptual) processes that occur at the beginning of an activity [6].

Fatigue is a recognised occupational problem [7,8]. Occupational fatigue is defined as a multidimensional state, ranging from acute to chronic [9]. It appears in individuals exposed through their work tasks, environment, and schedules to demands that exceed their ability, and can hinder their physical and cognitive skills [10,11]. In the scientific literature, there is a consensus that exhaustion can be used as an indicator of the psychological health of athletes [12] as well as coaches.

### 1.1. Coaching Role

Research on coaches has shown that their roles and responsibilities often affect their influence on athletes’ physical and psychological development, e.g., [13,14]. This view, which focuses on only one of the many elements that comprise the coach–athlete relationship, considers the general understanding of the profession and neglects the consideration of the wide spectrum of influence that coaches have [15].

Coaching requires complex decision making, player selection, and problem solving on a daily basis within significant time constraints [16]. Additionally, coaches must plan, prepare for training and/or competitions, and deal with boards of trustees and/or parents (in youth sport) and other external factors [17]. At the same time, coaches must manage their own emotional and physical state to perform optimally [18]. Due to the dynamic and ambiguous nature of the coaching profession, flexibility is considered essential, as this ambiguity often limits people’s ability to fulfil their roles [19]. The psychological and emotional aspects must be considered as well, as coaches work in a very competitive environment—even more so in elite categories—characterised by long working hours, high public exposure, and irregular and relatively short contracts [20]. Coaches may also be subject to highly competitive expectations, such as to obtain good results every day, and they depend on the athletes they lead having limited control over them [19]. The unregulated nature of the industry typically results in coaches working long hours (often with no scheduled regular days off) during periods of intense stress [21,22]. Fatigue can result directly from poor working conditions [23]. Many athletes experience periods of tiredness and exhaustion to the point of wanting to stop participating in sports or quitting [24]; coaches are no strangers to these thoughts, as they are under even greater pressure than athletes.

### 1.2. Fatigue and Coaching Work

Fatigue has a negative impact on commitment to a task where mental effort is required [25]. Resistance and fatigue are important aspects of team and player/athlete management in all competitive sports [26], and therefore, the coaching collective. In professional and university level sports, the health management of athletes is fundamental to the success of the team [26]. The coach’s health may be equally as important to this success, as the coach plays a key role in the planning and development of training and matches, deciding when and how to train, choosing which players will participate and which will sit on the bench, and influencing to whom players are signed. The presence of fatigue is associated with psychological, symptomatic disorders and decreased functional status [27]. Fatigue often appears in conjunction with depressive symptoms, anxiety, and sleep impairment and is associated with a poor state of subjective health perception and a low or decreased level of quality of life [28,29,30,31,32,33].

The amount and extent of fatigue symptoms and factors depend on characteristics of the sports activity, the individual, and the environment [34]. Contextual variables such as match location, the strength of the opposition, and match outcome affect subjective measures of well-being and fatigue differently [35].

Coaches, by the nature of their role, can significantly shape athletes’ experiences on and off the field [36]. From the point of view of emotional contagion, stressed and anxious coaches can easily mistreat their athletes; in turn, athletes may develop negative attitudes towards coaches [37]. The coach is an important member of the sport-related social environment with great potential to influence athletes’ psychological health outcomes [38].

The perception that the lack of staff cohesion contributes to mental fatigue in the elite sports environment is an interesting and novel finding [39]. The results of numerous studies suggest that coaches who invest in developing high-quality relationships with their athletes can optimize the sports experience, performance, and well-being of athletes [40,41]. Over the course of a season, athletes’ perceptions of their coach can fluctuate in both intensity and direction [42]; these perceptions likely influence their relationship with the coach.

Within the student athlete environment, coaches have been identified both as a potential source of pressure and support [43].

Mental fatigue and motivation have been shown to be related [44,45]. Fatigue can lead to inappropriate behaviour [46], absence from work [47], decreased job satisfaction and quitting [48].

Fatigue could negatively affect perseverance, planning [49], activity tracking [50,51,52], and a person’s ability to retrieve information [53]. Increased awareness among employers and employees of the impact of fatigue has led to the recognition of the need for information on the extent of fatigue at work and the risk factors involved in such cases [54].

### 1.3. Grit

Positive psychology has defined grit as the ability to persevere, to have passion, and to commit to achieving long-term goals regardless of adversity or challenge [55]. Robinson [56] described grit more clearly as the consistency of interest and perseverance in effort. Conceptually speaking, grit includes a specific and unique approach to reaching long-term higher-order goals [57].

Determination is a non-cognitive personality trait that is operationalised as a higher-order construct with two lower-order characteristics, perseverance in effort and consistency of interest [58,59]. Theoretically, these facets work together to influence an individual’s attitude and behaviour toward long-term goals [60]. People with greater determination show greater work commitment [61] and less counterproductive work behaviours [62]. In the United States, the construct of grit has been considered the new gold standard to predict personal and work-related success [63]. Several studies have corroborated that the Grit-S scale is a good measure to predict workplace retention [61] and depletion risks [64]. Grit has been extensively studied in various educational contexts, including its role in academic success [65,66,67]. However, more research is still needed in the workplace and in non-educational environments [68].

As the preceding review shows, coaches’ health may affect their interpersonal behaviour with athletes. Thus, understanding how to optimize coaches’ health is vital, and could also affect their relationship with athletes and result in an improvement of athletes and teams. The study of fatigue in relation to the type of contract is supported by the fact that those workers with the highest absenteeism and job dissatisfaction are those without permanent and/or precarious employment [69].

This study has the following objectives: (i) to identify the level of general fatigue (FG), physical fatigue (FF) and concentration/motivation (C/M) in sports coaches; (ii) to determine the level of grit, specifically consistency of interest (CI) and perseverance in effort (PE) in coaches; and (iii) to establish the possible effect of sex, age, marital status, employment contract, work dedication and grit on FG, FF and C/M in coaches.

## 2. Materials and Methods

This research uses a non-experimental design, since no variables are manipulated and can be described as cross-sectional because the data are collected at a given time through questionnaires. Further, this research is quantitative and uses a correlational approach because it seeks to determine relationships between study variables [70].

### 2.1. Participants

Participants for the sample were selected using a non-probabilistic convenience procedure [71] based on certain criteria (the objectives of the research project, the material and human resources available, and individuals’ availability). According to the inclusion criteria, eligible participants were required to coach a sport, either individual or collective; be over 18 years of age; and agree to participate voluntarily in the research. For this purpose, informed written consent was obtained from all participants.

The initial sample consisted of 362 participants. After eliminating those with incomplete questionnaires (7.46%, *n* = 27), 335 coaches remained. Women comprised 21.2% (*n* = 71) and 78.8% (*n* = 264) were men, with a mean age of 29.88 (SD = 9.97) years and a mean number of years as a coach of 3.97 (SD = 4.14). Regarding marital status, 52.2% (*n* = 175) were single, 44.8% (*n* = 150) were married or a couple, and the rest were in another situation. Regarding educational status, 42.6% (*n* = 142) had university studies, 54.1% (*n* = 180) had a bachelor’s degree or vocational training, and the remainder of the sample had primary studies. Regarding employment, 57.1% (*n* = 186) had an employment contract and the remainder had no contract. In relation to work dedication, 75% (*n* = 249) worked half-time, 17.5% (*n* = 58) worked part-time and the remainder worked full-time. Half-time means 4 h of work per day and part-time means those who work sporadically or less than 4 h per day every day they work.

### 2.2. Procedure

After contacting the managers of different sports clubs and receiving the appropriate permission, visits were made for the administration of the questionnaires. Participation was voluntary and anonymous. All participants signed informed consent in accordance with the principles of the Helsinki Declaration [72]. Approval was sought from the Ethics Committee of Research of the University of Murcia, which determined that the study, despite using human subjects, was observational and did not contain any sensitive information, and therefore did not require the approval of the committee.

### 2.3. Instrument

The Multidimensional Fatigue Inventory (MFI) of Smets et al. [73] was used to assess fatigue. The MFI is a valid and reliable tool that has been used in previous studies that measured fatigue in both the general and working populations [74,75,76]. This study used the Spanish adaptation of the MFI by Boada-Grau et al. [77], which has established validity and reliability. It is a 19 item self-report measure consisting of three factors/dimensions: general fatigue (FG), physical fatigue (FF), and concentration/motivation (C/M). Participants assess fatigue using a 7-point Likert scale to indicate to what extent a statement is true for them, ranging from 1 (*that is true*) to 7 (*no, that is not true*). In this study, the scale had the following Cronbach’s alpha (α): FG, α = 0.89; FF, α = 0.93; and C/M, α = 0.91.

The Grit-S scale [59] aims to measure the levels of perseverance and passion for achieving long-term goals. This study used the Spanish-adapted version of Fernández-Martín et al. [78]. The eight-item Grit-S scale uses a 5-point Likert-type scale ranging from 1 (*does not describe me at all*) to 5 (*totally describes me*). The scale consists of two subscales. Consistency of interest (CI) is composed of four items, which are reversed following Thomas et al. [79] (e.g., ‘New ideas and projects sometimes distract me from previous ideas and projects’). Perseverance in effort (PE) also has four items (e.g., ‘The setbacks do not discourage me’). The CI subscale refers to the tendency not to change objectives and interests frequently; the PE subscale assesses the tendency to work hard even in the face of setbacks. In this study, CI received a Cronbach’s alpha value of 0.9, while PE received a value of 0.91.

### 2.4. Statistical Analysis

A descriptive analysis of qualitative variables was performed with absolute and relative frequencies. An ANOVA was used for group-to-group comparisons and multiple regression was used to determine the effects of the different variables on dimensions of fatigue and grit. In addition, intraclass agreement coefficients (ICCs) were calculated to determine inter-rater reliability. In this case, the clinical significance was defined as poor for an ICC below 0.4, fair to good for 0.40–0.75, and excellent for 0.75 or higher [80]. Relationships between fatigue and grit and their dimensions were assessed using Pearson’s product–moment correlation. According to Hopkins et al. [81], the magnitude of correlation coefficients was considered as trivial (r < 0.1), small (from 0.1 to <0.3), moderate (from 0.3 to <0.5), large (from 0.5 to <0.7), very large (from 0.7 to <0.9), nearly perfect (r from 0.9 to <1) and perfect (r = 1). Statistical analyses were performed with the SPSS version 25.0 software program for Windows (IBM; Armonk, NY, USA). A significance level of *p* < 0.05 was set for all analyses.

## 3. Results

Table 1 shows the means, standard deviation, Cronbach’s alpha, and the correlation matrix between the MFI dimensions and Grit-S subscales. FG was positively correlated with CI, and negatively correlated with C/M and PE; these relationships were significant. FF was positively correlated with C/M and negatively correlated with PE; both relationships were significant. C/I was negatively and significantly correlated with PE.

No statistically significant gender differences were found for either the MFI or Grit-S scales. Men obtained higher values in FF (29.52, SD = 4.16), C/M (28.72, SD = 3.99) and PE (3.95, SD = 0.52). Women achieved higher scores in FG (22.38, SD = 8.74) and CI (2.95, SD = 0.81).

In terms of labour dedication, no statistically significant differences were obtained. Coaches with a part-time workday indicated higher FG values (22.27, SD = 8.96), while those who worked full-time showed higher scores in FF (30.88, SD = 3.91); C/M (28.44, SD = 3.28); and PE (4.08, SD = 0.44). Those who worked part-time or full-time achieved the highest score in CI (2.94, SD = 0.86). No significant differences were found in relation to scales (MFI/Grit-S) and sport type (collective/individual). Coaches of collective sports obtained higher values of FG (22.37, SD = 8.98); FF (29.55, SD = 4.48); CI (3.01, SD = 0.78); and PE (3.95, SD = 0.53). Coaches of individual sports scored higher in C/M (28.16, SD = 4.38). In regard to years of coaching experience, no statistically significant differences were found.

Table 2 depicts the results of an ANOVA analysis examining the relationship of marital status and MFI and Grit-S scores. Statistically significant differences were found in C/M. Coaches who were single or married/a couple scored lower than coaches with a different marital status. No other significant differences were found. Coaches who were single indicated the highest FG values, while coaches who were married/a couple obtained the highest values in FF and CI. Coaches in other marital arrangements received the highest scores in PE.

Table 3 depicts MFI and Grit-S scores in relation to whether coaches had a contract, as well as the results of the Student’s *t*-tests performed to determine statistically significant differences between those coaches with contracts and those without. The results showed that coaches without contracts scored higher in FG, CI and PE, while coaches with contracts scored higher in C/M and FF; the value for FF was statistically significant (*p* = 0.045).

Table 4 depicts MFI and Grit-S scores in relation to the age category (child/youth/senior) of the coaches’ team. The dimension of FF received the highest score in all three categories, and decreased from child (29.37, SD = 4.47) to juvenile (29.14, SD = 4.30) to senior (29.03, SD = 4.81) level. Statistically significant differences were found in the CI between coaches working with the youth category and those who worked with the senior category or with children (*p* = 0.014). Coaches who trained children received the highest CI values (3.06, SD = 0.78). The remaining dimensions did not show statistically significant differences.

A multivariate linear regression was performed to determine the possible effects on FG of sex (male vs. female), age, marital status (married vs. single and other vs. single), employment contract (yes vs. no), work dedication (half-time vs. part-time and full-time vs. part-time) and Grit-S scores (CI and PE); see Table 5. The model was statistically significant, *F* (9, 311) = 9.64, *p* < 0.001, and explained 21.8% of the variance in FG. None of the sociodemographic variables were statistically significant. Regarding the Grit-S scores, the CI subscale showed a statistically significant effect, such that coaches with higher CI values had greater overall fatigue. PE scores showed a statistically significant negative effect, such that high levels of PE were associated with low levels of overall fatigue. Additionally, for the FF, the model was statistically significant: *F* (9, 311) = 6.55; *p* < 0.001, explaining 20.3% of the variance in physical fatigue. None of the sociodemographic variables were statistically significant. Regarding Grit-S scores, both the CI and PE subscales showed a statistically significant effect, such that coaches with higher levels of CI and PE had greater FF. Similarly, for C/M, the model was statistically significant, *F* (9, 311) = 11.51; *p* < 0.001) and explained 25% of the variance in concentration. Marital status showed statistically significant differences, as did the perseverance subscale (*p* < 0.001). Thus, high PE values were associated with high C/M values.

## 4. Discussion

This study aimed to determine levels of general fatigue (FG), physical fatigue (FF), and concentration/motivation (C/M) in club sports coaches. Levels of the two components of grit, consistency (CI) and perseverance in effort (PE), were also examined. Finally, the possible effects of sex, age, marital status, employment contract, and work dedication relative to the dimensions of fatigue and grit were investigated.

In relation to FG, the study sample indicated an average level, indicating that the coaches in the sample, despite the constraints of their work, complex decision making with significant time constraints [16], managing their own emotional and physical state to perform at their best, [18] do not show severe symptoms of general fatigue. These data are in line with the results of Watt et al. [82] in the general Danish population without somatic disease. Although this level differs from that found by Bazazan et al. [83] in a sample of petrochemical employees, these employees were subject to turnicity, working outside of regular hours.

However, this result differs from the findings of Shahril Abu Hanifah and Ismail [84], who found FG to be higher in a majority of Malaysian electronics workers, as well as Remmen et al. [85], who found FG to be higher in Danish fishermen. As labour demands increase, effort must be mobilised to maintain performance levels, which is associated with physiological and psychological costs [86]. Fatigue can be both mental and physical. However, in real situations, a combination of symptoms may occur in varying proportions depending on the nature of the tasks in question [87].

The FF dimension of fatigue obtained the highest mean value in the current study, as coaches have very long working hours with intense periods of stress [21,22] and the need to perform well every day [20], all in a highly competitive environment with relatively short contracts and high public exposure [21]. These data are congruent with Bazazan et al. [88] in petrochemical plant operators, Jalilian et al. [89] in a study of Iranian nurses and partially with Tirviené et al. [90], who analysed duty nurses. These are values that may be affected by job stress, which helps to reduce worker performance [89].

As for sex, no significant differences were found in this study in the dimensions of fatigue, in line with previous studies [73,91,92]. In contrast, Engberg et al. [93] detected statistically significant differences in Sweden’s general population. Women in the current study had slightly higher mean FG scores than men, supporting previous studies that found fatigue was more common in women [74,82,94,95,96,97,98]; fatigue also has been closely related to the female gender [99]. Men in the current study showed higher mean FF values, in line with the findings of Guo et al. [100] in automobile factory employees. A cause that may be amplified by the social role of women, with the additional burden of family and household chores in addition to work outside the home.

In relation to marital status, single coaches obtained higher mean values in FG, in contrast to Jason et al. [96], who found that separated coaches scored the highest in this subscale. Married/couple coaches had lower FG scores, congruent with a number of studies [82,94]. Marriage and cohabitation status seem to be a positive factor in alleviating fatigue, possibly related to the support of family and friends [101]. Supportive family and personal relationships have been identified as important coping mechanisms to reduce fatigue [102]. Marriage has also been found to be a protective factor of fatigue, as married workers may have better living conditions that influence their feelings of fatigue [103]. However, Fang et al. [104] found high fatigue levels in married nurses.

Coaches with a marital status other than single/married/couple scored higher in C/M, supporting results by Shahril Abu Hanifah and Ismail [84], who found reduced motivation in people who were divorced. In the general German population, Kocavelent et al. [105] found that being separated or divorced increased the likelihood of experiencing fatigue and stress, which may be due to the combination of work outside the home and work at home, where the majority of the work is carried out by women, accounting for more than 90% of the total.

Coaches’ years of experience did not show a relationship to the dimensions of fatigue, consistent with previous studies [74,106]. This is an element that can be cushioned by the experience of coaches learning to cope with the negative effects of their professional role.

Nor was a significant relationship identified between coaches’ years of experience and concentration/motivation, corroborating the results of Zakeri et al. [107].

This variable is crucial in the work of the coach, as on numerous occasions they are hired when teams are not doing well and they are the ones who must motivate and raise the morale of the new team they are managing, or when they are already in charge and there is a defeat, the coaches are the psychological engine that reactivates that lost motivation or concentration.

The mean Grit-S scores of the coaches in this study are consistent with Musso et al. [108] and means for adults 25 years and older found by Duckworth et al. [109]. Coaches in this study did not demonstrate significantly more or less determination than the general population. Further, no statistically significant differences were found when examining the relationship of grit scores and sex, in line with previous studies [110,111,112]. However, men obtained higher values in perseverance, while women scored higher in consistency.

The study results presented should be considered in relation to methodological limitations. While cross-sectional correlational designs are common in research, this design has some restrictions, such as the inability to establish causal relationships. Further, collecting self-reported data, while a typical practice in studies, can lead to a bias in participants’ responses, exacerbate common variance and artificially increase correlations between variables [113]. Further, it is well known that subjective reports are affected by conscious bias. The study sample was composed of Spanish workers, who have their own cultural characteristics; therefore, the results cannot be generalised to other populations. It would be interesting to conduct intercultural or transnational studies to see if the results of our work are similar to those found in other countries. These limitations should be considered in future research, which may benefit from using more sophisticated designs.

## 5. Conclusions

The main findings of the study are as follows. Male coaches obtained higher values in FF, C/M and PE, while women scored higher in FG and CI. These elements make us see that men are more affected by the physical factor, being stronger in motivation/concentration and perseverance in the effort, perhaps because of this assumption of the role of the trainer, imbricated in the sporting sphere. Single coaches received the highest values in FG, while coaches who were married/couples obtained the highest scores in FF and CI. Having one marital status or the other tips the balance towards general fatigue in singles and physical fatigue in married couples, an important factor to analyse and bear in mind in the day-to-day work of the trainer.

Coaches without contracts had higher FG, CI and PE scores, while those with contracts achieved higher scores in C/M and FF. The responsibility is heavy, and even more so for the team leader, the coach, who must have answers to all the situations that occur, when there are victories, failures, good or bad atmosphere in the dressing room, friction between athletes, professional egos, and relations with the fans; there are multiple variables that they must manage and many of them indirectly, which generates more uncertainty.

The population the coaches worked with influenced FF scores, with coaches of children obtaining the highest FF scores, followed by coaches of youth and senior-level teams. Coaches with higher CI scores had higher FG, while high levels of PE were associated with low FG levels. Greater levels of CI and PE were associated with higher FF values. High PE values were also associated with high C/M values.

Coaches who have perseverance in effort are less fatigued, while those who indicate consistency of interest are more fatigued.

## Figures and Tables

**Table 1:** Means, standard deviations, reliability and correlations of the MFI and Grit-S scores.

	Mean (SD)	Cronbach’s α	General Fatigue	Physical Fatigue	Concentration	Consistency
**General fatigue**	22.07 (8.77)	0.897	1			
**Physical fatigue**	29.34 (4.42)	0.937	−0.107	1		
**Concentration**	28.08 (4.02)	0.917	−0.342 **	0.466 **	1	
**Consistency**	2.94 (0.83)	0.9	0.395 **	0.085	−0.233 *	1
**Perseverance**	3.93 (0.53)	0.912	−0.254 **	0.356 **	0.474 **	−0.168 **

* *p* < 0.05; ** *p* < 0.01.

**Table 2:** ANOVA results and means of MFI and Grit-S scores relative to marital status.

	Marital Status, Mean (SD)			ANOVA		η^2^
	Single	Married/ A Couple	Other	*F* (2.332)	*p*-Value	
**General fatigue**	22.41 (8.18)	21.63 (9.17)	20.10 (9.50)	0.57	0.569	0.003
**Physical fatigue**	29.11 (4.50)	29.65 (4.38)	28.30 (3.83)	0.87	0.419	0.005
**Concentration**	27.69 (4.36) a	28.34 (3.60) a	30.80 (2.78) b	3.46	**0.033**	0.021
**Consistency**	2.97 (0.82)	2.92 (0.81)	2.80 (1.21)	0.31	0.735	0.002
**Perseverance**	3.93 (0.54)	3.93 (0.50)	4.10 (0.58)	0.52	0.596	0.003

a,b: two-to-two column comparisons.

**Table 3:** Means and *t*-test results of coaches’ contracts and MFI and Grit-S scores.

	Contract, Mean (SD)		Difference Half	*t*-Test		d
	No	No		*t* (324)	*p*-Value	
**General fatigue**	22.87 (9.57)	21.13 (7.52)	1.74	1.84	0.067	0.21
**Physical fatigue**	28.74 (4.48)	29.73 (4.32)	−0.99	−2.01	**0.045**	−0.23
**Concentration**	27.80 (3.93)	28.23 (4.10)	−0.43	−0.95	0.345	−0.11
**Consistency**	2.99 (0.88)	2.89 (0.78)	0.11	1.14	0.254	0.13
**Perseverance**	3.94 (0.55)	3.92 (0.51)	0.03	0.44	0.662	0.05

SD: standard deviation. d: Cohen’s effect size.

**Table 4:** ANOVA results and means of MFI and Grit-S scores relative to training category.

	Category, Mean (SD)			ANOVA		η^2^
	Child	Youth	Senior	*F* (2.332)	*p*-Value	
**General fatigue**	22.07 (8.77)	22.27 (8.39)	22.03 (8.74)	0.02	0.98	0.000
**Physical fatigue**	29.37 (4.47)	29.14 (4.30)	29.03 (4.81)	0.16	0.856	0.001
**Concentration**	27.96 (4.00)	27.70 (3.71)	28.29 (4.76)	0.39	0.675	0.003
**Consistency**	3.06 (0.78) a	2.74 (0.86) b	2.96 (0.86) a	4.37	**0.014**	0.028
**Perseverance**	3.91 (0.55)	3.94 (0.51)	3.93 (0.52)	0.16	0.853	0.001

a,b: two-to-two column comparisons.

**Table 5:** Linear regression model of variables and subscales on general fatigue, physical fatigue, and concentration.

	General Fatigue				Physical Fatigue				Concentration			
	B (SE)	Beta	*t*	*p*-Value	B (SE)	Beta	*t*	*p*-Value	B (SE)	Beta	*t*	*p*-Value
**Sex (male vs. female)**	0.97 (1.06)	0.046	0.91	0.364	−0.25 (0.56)	−0.023	−0.437	0.662	−0.76 (0.49)	−0.077	−1.55	0.122
**Age**	0.06 (0.05)	0.07	1.271	0.205	−0.04 (0.03)	−0.1	−1.759	0.08	−0.02 (0.02)	−0.041	−0.768	0.443
**Marital status**												
Married/couple vs. single	−1.02 (0.91)	−0.06	−1.122	0.263	0.65 (0.48)	0.075	1.345	0.179	0.81 (0.42)	0.101	1.919	0.056
Other vs. single	−1.80 (2.57)	−0.037	−0.7	0.484	−0.66 (1.36)	−0.027	−0.487	0.627	2.70 (1.19)	0.118	2.261	0.024
**Employment contract (yes vs. no)**	−1.06 (0.92)	−0.062	−1.149	0.251	0.82 (0.49)	0.094	1.681	0.094	0.56 (0.43)	0.069	1.308	0.192
**Work dedication**												
Half-time vs. part-time	−0.68 (1.19)	−0.03	−0.569	0.57	−0,14 (0.63)	−0.012	−0.217	0.828	−0.17 (0.55)	−0.017	−0.316	0.752
Full-time vs. part-time	−0.42 (1.66)	−0.013	−0.252	0.801	1.04 (0.87)	0.065	1.184	0.237	−0.34 (0.77)	−0.023	−0.437	0.662
**Consistency**	3.50 (0.52)	0.342	6.705	**<0.001**	0.80 (0.28)	0.153	2.894	**0.004**	−0.24 (0.24)	−0.05	−0.991	0.323
**Perseverance**	−3.84 (0.83)	−0.236	−4.614	**<0.001**	2.88 (0.44)	0.349	6.582	**<0.001**	3.48 (0.39)	0.453	9.032	**<0.001**

B: non-standardised regression coefficient. SE: typical error. Beta: standardised regression coefficient.

## Data Availability

Not applicable.

## References

[1] Barker L.M., Nussbaum M.A. (2011). The effects of fatigue on performance in simulated nursing work. Ergonomics.

[2] Mahdavi N., Dianat I., Heidarimoghadam R., Khotanlou H., Faradmal J. (2020). A review of work environment risk factors influencing muscle fatigue. Int. J. Ind. Ergon..

[3] Pedraz-Petrozzi B. (2018). Fatiga: Historia, neuroanatomía y características psicopatológicas. Una revisión de la Literatura. Rev. Neuro-Psiquiatr..

[4] Ferré A. (2018). Chronic fatigue syndrome and sleep disorders: Clinical associations and diagnostic difficulties. Neurología.

[5] Mueller-Schotte S., Bleijenberg N., Van der Schouw Y.T., Schuurmans M.J. (2016). Fatigue as a long-term risk factor for limitations in instrumental activities of daily living and/or mobility performance in older adults after 10 years. Clin. Interv. Aging.

[6] Yung M., Wells R.P. (2017). Documentar el patrón temporal del desarrollo de la fatiga. Trans. IISE Sobre Ergon. Ocup. Factores Hum..

[7] Ahsberg E. (2000). Dimensions of fatigue in different working populations. Scand. J. Psychol..

[8] Handcock P.A., Desmond P.A. (2008). Stress, Workload and Fatigue.

[9] Brzozowski S.L., Cho H., Knudsen É.N., Steege L.M. (2021). Predicting nurse fatigue from measures of work demands. Appl. Ergon..

[10] Barker L.M., Nussbaum M.A. (2011). Fatigue, performance and the work environment: A survey of registered nurses. J. Adv. Nurs..

[11] Barker L.M., Nussbaum M.A. (2013). Dimensions of fatigue as predictors of performance: A structural equation modeling approach among registered nurses. IIE Trans. Occup. Ergon. Hum. Factors.

[12] Gustafsson H., Lundkvist E., Podlog L., Lundqvist C. (2016). Conceptual confusion and potential advances in athlete burnout research. Percept. Mot. Ski..

[13] Iosard-Gautheur S., Guillet-Descas E., Lemyre P.N. (2012). A prospective study of the influence of perceived coaching style on burnout in high level young athletes: Using a self-determination theory perspective. Sport Psychol..

[14] Williams A.M., Reilly T. (2000). Talent identification and development in soccer. J. Sports Sci..

[15] Cote J., Gilbert W. (2009). An integrative definition of coaching effectiveness and expertise. Int. J. Sports Sci. Coach..

[16] Altfeld S., Kellmann M. (2015). Are German coaches highly exhausted? A study of differences in personal and environmental factors. Int. J. Sports Sci. Coach..

[17] Miller P.S., Salmela J.H., Kerr G. (2002). Coaches’ Perceived Role in Mentoring Athletes. Int. J. Sport Psychol..

[18] Thelwell R., Weston J.V., Greenless I.A., Hutchings N. (2008). Stressors of Elite Sports. J. Sports Sci..

[19] Jones R.L., Wallace M. (2005). Another bad day at the training ground: Coping with ambiguity in the coaching context. Sport Educ. Soc..

[20] Bentzen M., Lemyre P.N., Kentta G. (2016). Development of exhaustion for high performance coaches in association with workload and motivation: A person-centered approach. Psychol. Sport Exerc..

[21] Lyle J. (2002). Sports Coaching Concepts: A Framework for Coaches’ Behaviour.

[22] Robinson P.E. (2010). Foundations of Sports Coaching.

[23] Ahmed M.F., Sleem W.F., Kassem A.H. (2015). Effect of working condition and fatigue on performance of staff nurses at Mansoura University Hospital. J. Nurs. Health Sci..

[24] Raedeke T.D., Lunney K., Venables K. (2002). Understanding athletes burnout: Coach perspectives. J. Sport Behav..

[25] Van Cutsem J., Marcora S., De Pauw K., Bailey S., Meeusen R., Roelands B. (2017). The effects of mental fatigue on physical performance: A systematic review. Sports Med..

[26] Carrington P., Ketter D., Hurst A. Understanding fatigue and stamina management opportunities and challenges in wheelchair basketball. Proceedings of the 19th International ACM SIGACCESS Conference on Computers and Accessibility.

[27] Irvine D., Vincent L., Graydon J.E., Bubela N., Thomson L. (1994). The prevalence and correlates of fatigue in patients receiving treatment with chemotherapy and radiotherapy: A comparison with the fatigue experienced by healthy individuals. Cancer Nurs..

[28] Jacobsen P.B., Donovan K.A., Weitzner M.A. (2003). Distinguishing fatigue and depression in patients with cancer. Semin. Clin. Neuropsychiatry.

[29] Redeker N.S., Lev E.L., Ruggiero J. (2000). Insomnia, fatigue, anxiety, depression, and quality of life of cancer patients undergoing chemotherapy. Res. Theory Nurs. Pract..

[30] Roscoe J., Kaufman M., Matteson-Rusby S., Palesh O., Ryan J., Kohli S., Morrow G. (2007). Cancer-related fatigue and sleep disorders. Oncologist.

[31] Font E., Rodriguez E., Buscemi V. (2004). Fatiga, expectativas y calidad de vida en cáncer. Psicooncologia.

[32] Johansson S., Ytterberg C., Hillert J., Widen Holmqvist L., Von Koch L.A. (2008). A longitudinal study of variations in and predictors of fatigue in multiple sclerosis. J. Neurol. Neurosurg. Psychiatry.

[33] Stanton B.R., Barnes F., Silber E. (2006). Sleep and fatigue in multiple sclerosis. Mult. Scler. J..

[34] Knicker A.J., Renshaw I., Oldham A.R., Cairns S.P. (2011). Interactive processes link the multiple symptoms of fatigue in sport competition. Sports Med..

[35] Abbott W., Brownlee T.E., Harper L.D., Naughton R.J., Clifford T. (2018). The independent effects of match location, match result and the quality of opposition on subjective wellbeing in under 23 soccer players: A case study. Res. Sports Med..

[36] Bennie A., Apoifis N., Caron J., Falcao W., Marlin D., Bengoechea E.G., George E. (2017). A guide to conducting systematic reviews of coaching science research. Int. Sport Coach. J..

[37] Chen L.H., Tasi I.M. (2005). The relationship among Chinese paternalistic leadership, emotional impression and athletic burnout. Sports Exerc. Res..

[38] Russell S., Jenkins D., Rynne S., Halson S.L., Kelly V. (2019). What is mental fatigue in elite sport? Perceptions from athletes and staff. Eur. J. Sport Sci..

[39] Davis L., Jowett S., Lafrenière M.A. (2013). An attachment theory perspective in the examination of relational processes associated with coach-athlete dyads. J. Sport Exerc. Psychol..

[40] Jowett S., Felton L. (2014). Relationships and Attachments in Teams. Group Dynamics Advances in Sport and Exercise Psychology.

[41] Felton L., Jowett S. (2017). A self-determination theory perspective on attachment, need satisfaction and well-being in a sample of athletes: A longitudinal study. J. Clin. Sport Psychol..

[42] Ronkainen N.J., Ryba T.V., Littlewood M., Selänne H. (2018). ‘School, family and then hockey!’ Coaches’ views on dual career in ice hockey. Int. J. Sports Sci. Coach..

[43] Jowett S., Shanmugam V., Schinke R.J., McGannon K.R., Smith B. (2016). Relational coaching in sport: It’s psychological underpinnings and practical effectiveness. Routledge International Handbook of Sport Psychology.

[44] Abbott W., Brownlee T.E., Naughton R.J., Clifford T., Page R., Harper L.D. (2020). Changes in perceptions of mental fatigue during a season in professional under-23 English Premier League soccer players. Res. Sports Med..

[45] Boksem M.A., Meijman T.F., Lorist M.M. (2006). Mental fatigue, motivation and action monitoring. Biol. Psychol..

[46] Potter P., Deshields T., Divanbeigi J., Berger J., Cipriano D., Norris L., Olsen S. (2010). Compassion fatigue and burnout: Prevalence among oncology nurses. Clin. J. Oncol. Nurs..

[47] Da Rocha M.C.P., De Martino M.M.F. (2010). Stress and sleep quality of nurses working different hospital shifts. Rev. Esc. Enferm. Da USP.

[48] Kuerer H.M., Eberlein T.J., Pollock R.E., Huschka M., Baile W.F., Morrow M., Michelassi F., Singletary S.E., Novotny P., Sloan J. (2007). Career satisfaction, practice patterns and burnout among surgical oncologists: Report on the quality of life of members of the Society of Surgical Oncology. Ann. Surg. Oncol..

[49] Van der Linden D., Frese M., Meijman T.F. (2003). Mental fatigue and the control of cognitive processes: Effects on perseveration and planning. Acta Psychol..

[50] Pattyn N., Neyt X., Henderickx D., Soetens E. (2008). Psychophysiological investigation of vigilance decrement: Boredom or cognitive fatigue?. Physiol. Behav..

[51] Shallice T., Stuss D.T., Alexander M.P., Picton T.W., Derkzen D. (2008). The multiple dimensions of sustained attention. Cortex.

[52] Cook D.B., O’Connor P.J., Lange G., Steffener J. (2007). Functional neuroimaging correlates of mental fatigue induced by cognition among chronic fatigue syndrome patients and controls. Neuroimage.

[53] Watanabe Y., Watanabe Y., Evengård B., Natelson B.H., Jason L.A., Kuratsune H. (2008). Preface and mini-review: Fatigue science for human health. Fatigue Science for Human Health.

[54] Kant I.J., Bültmann U., Schröer K.A.P., Beurskens A.J., Van Amelsvoort L.G., Swaen G.M. (2003). An epidemiological approach to study fatigue in the working population: The Maastricht Cohort study. Occup. Environ. Med..

[55] Duckworth A. (2016). Grit: The Power of Passion and Perseverance.

[56] Robinson W.L. (2015). Grit and Deomgraphic Characteristics Associated with Nursing Student Course Engagement.

[57] Arco-Tirado J.L., Fernández-Martín F.D., Hoyle R.H. (2018). Development and validation of a Spanish version of the Grit-S scale. Front. Psychol..

[58] Duckworth A.L., Peterson C., Matthews M.D., Kelly D.R. (2007). Grit: Perseverance and passion for long-term goals. J. Pers. Soc. Psychol..

[59] Duckworth A.L., Quinn P.D. (2009). Development and validation of the short grit scale (grit-s). J. Personal. Assess..

[60] Fosnacht K., Copridge K., Sarraf S.A. (2019). How valid is grit in the postsecondary context? A construct and concurrent validity analysis. Res. High. Educ..

[61] Eskreis-Winkler L., Shulman E.P., Beal S.A., Duckworth A. (2014). The grit effect: Predicting retention in the military, the workplace, school and the marriage. Front. Psychol..

[62] Ceschi A., Sartori R., Dickert S., Constantini A. (2016). Grit or honestyhumility? New insights into the moderating role of personality between the health impairment process and counterproductive work behaviour. Front. Psychol..

[63] Berkowitz P. ‘Grit’, by Angela Duckworth. https://www.nytimes.com/2016/05/08/books/review/grit-by-angeladuckworth.html.

[64] Salles A., Cohen G.L., Mueller C.M. (2014). The relationship between grit and resident well-being. Am. J. Surg..

[65] Duckworth A.L., Kirby T.A., Tsukayama E., Berstein H., Ericsson K. (2011). Deliberate Practice Spells Success: Why Grittier Competitors Triumph. Soc. Psychol. Personal. Sci..

[66] Cross T. (2014). The Gritty: Grit and Non-Traditional Doctoral Student Success. J. Educ. Online.

[67] Ivcevic Z., Brackett M. (2014). Predicting school success: Comparing Conscientiousness, Grit, and Emotion Regulation Ability. J. Res. Personal..

[68] Burkhart R.A., Thoely R.M., Guinto D., Yeo C.J., Chojnacki K.A. (2014). Grit: A marker of residents at risk for attrition?. Surgery.

[69] Benavides F.G., Benach J., Román C. (1999). Tipos de empleo y salud: Análisis de la Segunda Encuesta Europea de Condiciones de Trabajo. Gaceta Sanit..

[70] Namakforoosh M. (2005). Metodología de la Investigación.

[71] Pérez V.D. (2016). Procedimientos de Muestreo y Preparación de la Muestra.

[72] Asociación Médica Mundial (WMA) (2013). Declaración de Helsinki de la AMM: Principios Éticos para la Investigación Médica en Seres Humanos.

[73] Smets E.M., Garssen B., Bonke B., De Haes J.C. (1995). The Multidimensional Fatigue Inventory (MFI): Phychometric qualities of an instrument to assess fatigue. J. Psychosom. Res..

[74] Schwarz R., Krauss O., Hinz A. (2003). Fatigue in the general population. Oncol. Res. Treat..

[75] Lin J., Brimmer D.J., Maloney E.M., Nyarko E., BeLue R., Reeves W.C. (2009). Further validation of the Multidimensional Fatigue Inventory in a US adult population sample. Popul. Health Metr..

[76] Bazazan A., Rasoulzadeh Y., Dianat I., Safaiyan A., Mombeini Z., Shiravand E. (2014). Demographic factors and their relation to fatigue and mental disorders in 12-hour petrochemical shift workers. Health Promot. Perspect..

[77] Boada-Grau J., Merino-Tejedor E., Gil-Ripoll C., Segarra-Pérez G., Vigil-Colet A. (2014). Spanish adaptation of the Multidimensional Fatigue Inventory to the work environment. Univ. Psychol..

[78] Fernández-Martín F.D., Arco-Tirado J.L., Soriano-Ruíz M. (2018). Perseverance and passion for achieving long-term goals: Transcultural adaptation and validation of the Grit-S scale. Rev. De Psicol. Soc..

[79] Tomás J.M., Galiana L., Hontangas P., Oliver A., Sancho P. (2013). Evidencia acumulada sobre los efectos de método asociados a ítems invertidos. Psicológica.

[80] Fleiss J.L. (1986). The Design and Analysis of Clinical Experiments.

[81] Hopkins W.G., Marshall S.W., Batterham A.M., Hanin J. (2009). Progressive statistics for studies in sports medicine and exercise science. Med. Sci. Sports Exerc..

[82] Watt T., Groenvold M., Bjorner J.B., Noerholm V., Rasmussen N.A., Bech P. (2000). Fatigue in the Danish general population. Influence of sociodemographic. factors and disease. J. Epidemiol. Community Health.

[83] Bazazan A., Rasoulzadeh Y., Dianat I., Safaiyan A., Mombeini Z. (2019). Occupational fatigue and mental health complaints among 8-hour shift workers of petrochemical industries in Iran. Work.

[84] Shahril Abu Hanifah M., Ismail N. (2020). Fatigue and its associated risk factors: A survey of electronics manufacturing shift workers in Malaysia. Fatigue Biomed. Health Behav..

[85] Remmen L.N., Herttua K., Riss-Jepsen J., Berg-Beckhoff G. (2017). Fatigue and workload among Danish fishermen. Int. Marit. Health.

[86] Hakanen J.J., Bakker A.B., Schaufeli W.B. (2006). Burnout y compromiso laboral entre docentes. J. Sch. Psychol..

[87] Techera U., Hallowell M., Stambaugh N., Littlejohn R. (2016). Causes and consequences of occupational fatigue: Meta-analysis and systems model. J. Occup. Environ. Med..

[88] Bazazan A., Dianat I., Rastgoo L., Mombeini Z. (2018). Factors associated with mental health status of hospital nurses. Int. J. Ind. Ergon..

[89] Jalilian H., Shouroki F.K., Azmoon H., Rostamabadi A., Choobineh A. (2019). Relationship between Job Stress and Fatigue Based on Job Demand-control-support Model in Hospital Nurses. Int. J. Prev. Med..

[90] Tirvienė G., Spirgienė L., Šimatonienė V. (2020). Fatigue among intensive care unit nurses. Nurs. Educ. Res. Pract..

[91] Abdali N., Nobahar M., Ghorbani R. (2020). Evaluation of emotional intelligence, sleep quality, and fatigue among Iranian medical, nursing, and paramedical students: A cross-sectional study. Qatar Med. J..

[92] Fazli Z., Parizi H.G., Abadi M., Kouhnavard B. (2020). Investigating the Relationship between Anxiety and Depression with Fatigue and Job Involvement among Employees in a Copper Smelter. Int. J. Occup. Hyg..

[93] Engberg I., Segerstedt J., Waller G., Wennberg P., Eliasson M. (2017). Fatigue in the general population-associations to age, sex, socioeconomic status, physical activity, sitting time and self-rated health: The northern Sweden MONICA study 2014. BMC Public Health.

[94] Basu N., Yang X., Luben R.N., Whibley D., Macfarlane G.J., Wareham N.J., Khaw K., Myin P.K. (2016). Fatigue is associated with excess mortality in the general population: Results from the EPIC-Norfolk study. BMC Med..

[95] Fieo R.A., Mortensen E.L., Lund R., Avlund K. (2014). Assessing fatigue in late-midlife: Increased scrutiny of the multiple fatigue inventory-20 for communitydwelling subjects. Assessment.

[96] Jason L.A., Jordan K.M., Richman J.A., Rademaker A.W., Huang C.F., Mccready W., Frankenberry E.L. (1999). A community-based study of prolonged fatigue and chronic fatigue. J. Health Psychol..

[97] Hernández J.L., Serratos J.N., Alcaraz J.L.G., Maldonado A.A. (2017). Assessment of workload, fatigue, and musculoskeletal discomfort among computerized numerical control lathe operators in Mexico. IISE Trans. Occup. Ergon. Hum. Factors.

[98] Rose D.M., Seidler A., Nübling M., Latza U., Brähler E., Klein E.M., Beutel M.E. (2017). Associations of fatigue to work-related stress, mental and physical health in an employed community sample. BMC Psychiatry.

[99] Äkerstedt T., Knutsson A., Westerholm P., Theorell T., Alfredsson L., Kecklund G. (2004). Mental fatigue, work and sleep. J. Psychosom. Res..

[100] Guo F., Wang T., Ning Z. (2017). Subjective measures of work-related fatigue in automobile factory employees. Work.

[101] Xu N., Zhao S., Xue H., Fu W., Liu L., Zhang T., Huang R., Zhang N. (2017). Associations of perceived social support and positive psychological resources with fatigue symptom in patients with rheumatoid arthritis. PLoS ONE.

[102] Samaha E., Lal S., Samaha N., Wyndham J. (2007). Psychological, lifestyle, and coping contributors to chronic fatigue in shift-worker nurses. J. Adv. Nurs..

[103] Jing M.J., Wang J.J., Lin W.Q., Lei Y.X., Wang P.X. (2015). A community-based cross-sectional study of fatigue in middleaged and elderly women. J. Psychosom. Res..

[104] Fang J., Kunaviktikul W., Olson K., Chontawan R., Kaewthummanukul T. (2008). Factors influencing fatigue in Chinese nurses. Nurs. Health Sci..

[105] Kocavelent R.D., Hinz A., Brahler E., Klapp B.F. (2011). Determinants of fatigue and stress. BMC Res. Notes.

[106] Karimi A., Honarbakhsh M. (2016). Dimensions of Occupational Fatigue in Heavy Vehicle Drivers. J. Maz. Univ. Med. Sci..

[107] Zakeri N., Sarafraz M., Pishdar M., Jamali Z., Maryam M. (2016). Investigating the factors involved in job satisfaction of IT and medical record employees in university hospitals of Bandar Abbas in 2015. J. Mod. Med. Inf. Sci..

[108] Musso M., Tatum D., Hamer D., Hammarlund R., Son L., McMahon P. (2019). The relationship between grit and resilience in emergency medical service personnel. Ochsner J..

[109] Sigmundsson H., Haga M., Hermundsdottir F. (2020). Passion, grit and mindset in young adults: Exploring the relationship and gender differences. New Ideas Psychol..

[110] Terry D., Peck B. (2020). Academic and clinical performance among nursing students: What’s grit go to do with it?. Nurse Educ. Today.

[111] Vainio M.M., Daukantaitė D. (2016). Grit and different aspects of well-being: Direct and indirect relationships via sense of coherence and authenticity. J. Happiness Stud..

[112] Spector P.E. (2006). Method variance in organizational research truth or urban legend?. Organ. Res. Methods.

[113] Baldwin W., Stone A.A., Turkkan J.S., Bachrach C.A., Jobe J.B., Kurtzman H.S., Cain V.S. (2000). Information no One Else Knows: The Value of Self-Report. The Science of Self-Report. Implications for Research and Practice.

